# Supplementary material for: Distinct CCK-positive SFO neurons are involved in persistent or transient suppression of water intake
Source: Nat Commun. 2020 Nov 10;11:5692. doi: 10.1038/s41467-020-19191-0 (PMC7655816; doi:10.1038/s41467-020-19191-0)
Supplement: Supplementary file 1 — Supplementary Information [file 41467_2020_19191_MOESM1_ESM.pdf]

Supplementary Information

“Distinct CCK-positive SFO neurons are involved in persistent or transient suppression of water intake”

Matsuda et al.

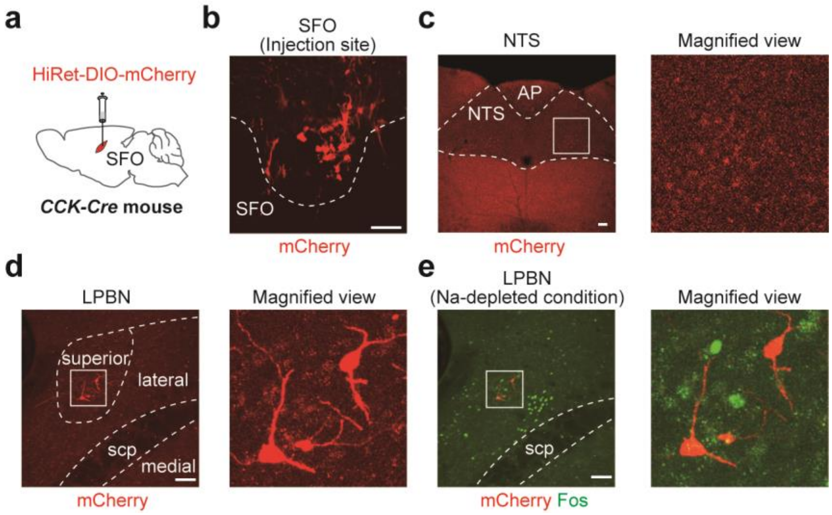

Supplementary Figure 1. CCK-positive neurons in the NTS and LPBN are not involved in the increase of CCK into the SFO under the Na-depleted condition.
(**a**) Injection of HiRet-DIO-mCherry into the SFO of the *CCK-Cre* mouse. (**b**–**d**) Immunohistochemical staining of mCherry in the SFO (**b**), NTS (**c**), and superior subnucleus of LPBN (**d**). (**e**) Immunohistochemical staining of mCherry and Fos in the LPBN under the Na-depleted condition. Several cells in the superior subnucleus of the LPBN were retrogradely labeled from the SFO, but were Fos-negative under the Na-depleted condition. These immunohistochemical images in **b**–**e** were reproduced from more than three independent mice. Scale bars, 50 μm.

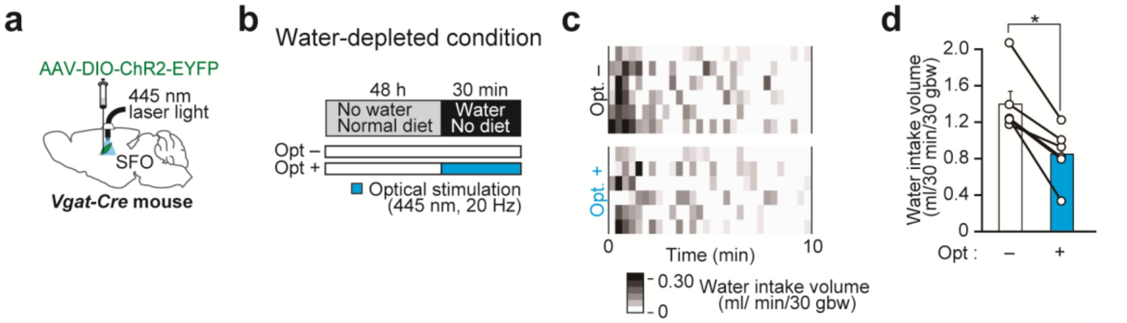

Supplementary Figure 2. Optical activation of GABAergic neurons in the SFO reduced water intake under the water-depleted condition.

(**a**) Injection of AAV-DIO-ChR2-EYFP into the SFO of the *Vgat*-Cre mouse. (**b**) Experimental protocol for the optical excitation of GABAergic neurons in the SFO under the water-depleted condition. We referred to a previous study[26] to perform this experiment. (**c**) Grayscale heat maps of water intake by individual mice with or without the optical stimulation. (**d**) Summary of water intake with or without the optical stimulation (n = 6 mice each; W = 21, P = 0.0313). *P < 0.05; two-sided Wilcoxon's signed rank tests. Data show the mean ± s.e.m.

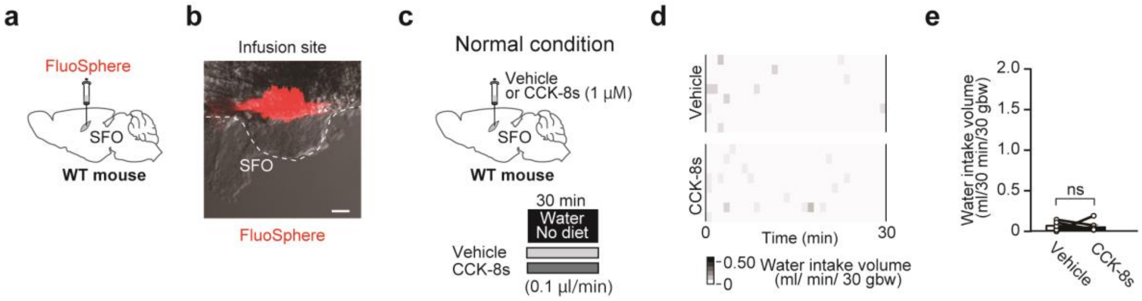

Supplementary Figure 3. Confirmation of the injection site of CCK-8s and effects of the injection of CCK-8s on water intake under the normal condition.

(**a**) Injection of CCK-8s into the SFO of WT mice together with FluoSphere-conjugated Alexa555. (**b**) Representative fluorescent image of FluoSphere in the SFO just after the injection. This image was reproduced from more than three independent mice. Scale bar, 50 μm. (**c**) Injection of CCK-8s (1.0 μM) into the SFO, and the experimental protocol under the normal condition. (**d**) Grayscale heat maps of water intake by individual mice received vehicle or CCK-8s. (**e**) Summary of data on water intake (n = 8 mice each; W = 21, P = 0.2968). ns, not significant; two-sided Wilcoxon's signed rank tests. Data show the mean ± s.e.m.

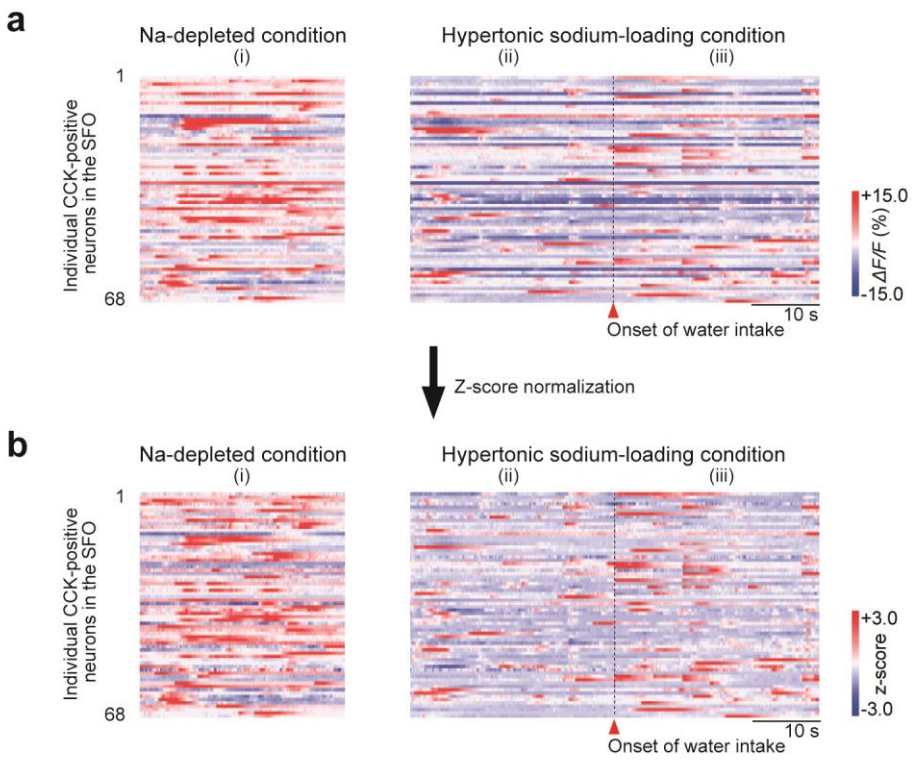

Supplementary Figure 4. *In vivo* activities of individual CCK-positive neurons in the SFO measured under the Na-depleted condition or hypertonic sodium-loading condition using a miniature fluorescent microscope.

(**a**) Raster plots of calcium responses (*ΔF/F*) in individual CCK-positive neurons during each condition (n = 68 neurons in 2 mice). (**b**) Normalized plots were individually calculated from the original activities of CCK-positive neurons in **a**. These plots were shown in Fig. 3e.

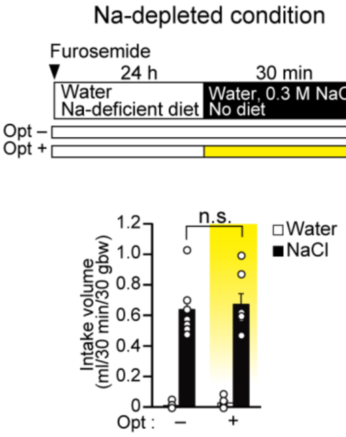

Supplementary Figure 5. The optical silencing of CCK-positive neurons in the SFO does not affect salt intake under the Na-depleted condition.

Upper; Experimental protocol for the two-bottle test (pure water and 0.3 M NaCl) with (opt+) or without (opt–) the optical silencing (wavelength, 577 nm) of eNpHR3.0-expressing CCK neurons in the SFO of the CCK-Cre mouse (Fig. 4a) under the Na-depleted condition. Lower; Summary of water and salt solution intakes under the Na-depleted condition with or without the optical silencing of CCK-positive neurons in the SFO (n = 7 mice each; $W = 10$, $P = 0.578$). bw, body weight; ns, not significant; two-sided Wilcoxon's signed rank tests. Data represent the mean ± s.e.m.

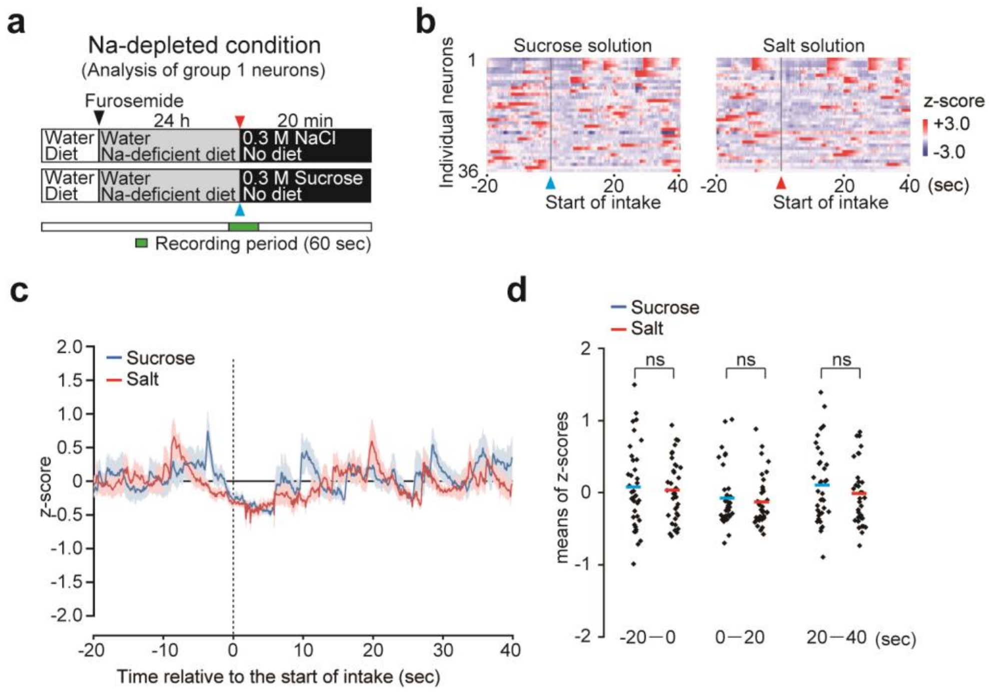

Supplementary Figure 6. Salt or sucrose intake under the Na-depleted condition does not rapidly affect the activity of group 1 CCK-positive neurons.

(**a**) Experimental protocol for Ca^{2+} imaging of group 1 CCK-positive neurons during drinking behaviors. (**b**) Raster plots of z-scored calcium responses in individual CCK-positive neurons while drinking 0.3 M NaCl or 0.3 M sucrose under the Na-depleted condition (n = 36 neurons in two mice). (**c**) Averages of calcium responses during access to the 0.3 M NaCl (or 0.3 M Sucrose) bottle. Shaded areas in red and blue indicate error bars. (**d**) Dot plot of the mean z-scores of individual CCK-positive neurons during each 20-sec interval at the three temporal positions indicated below (n = 36 neurons in two mice; $P_{(-20)} = 0.755$; $P_{(0)} = 0.599$; $P_{(20)} = 0.407$). ns, not significant; two-sided paired Student's *t*-tests. Data represent the mean ± s.e.m.

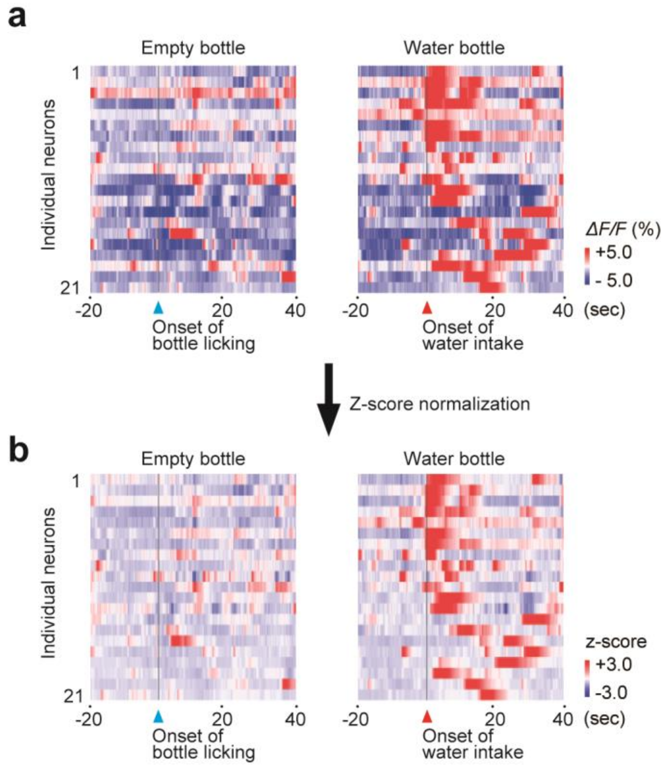

Supplementary Figure 7. *In vivo* activities of individual CCK-positive neurons measured at the onset of bottle licking or water intake using a miniature fluorescent microscope.

(**a**) Raster plots of calcium responses (*ΔF/F*) in individual CCK-positive neurons in the SFO during each condition (n = 21 neurons in 2 mice). (**b**) Normalized plots were individually calculated from the original activities of CCK-positive neurons in **a**. These plots were shown in Fig. 6b.

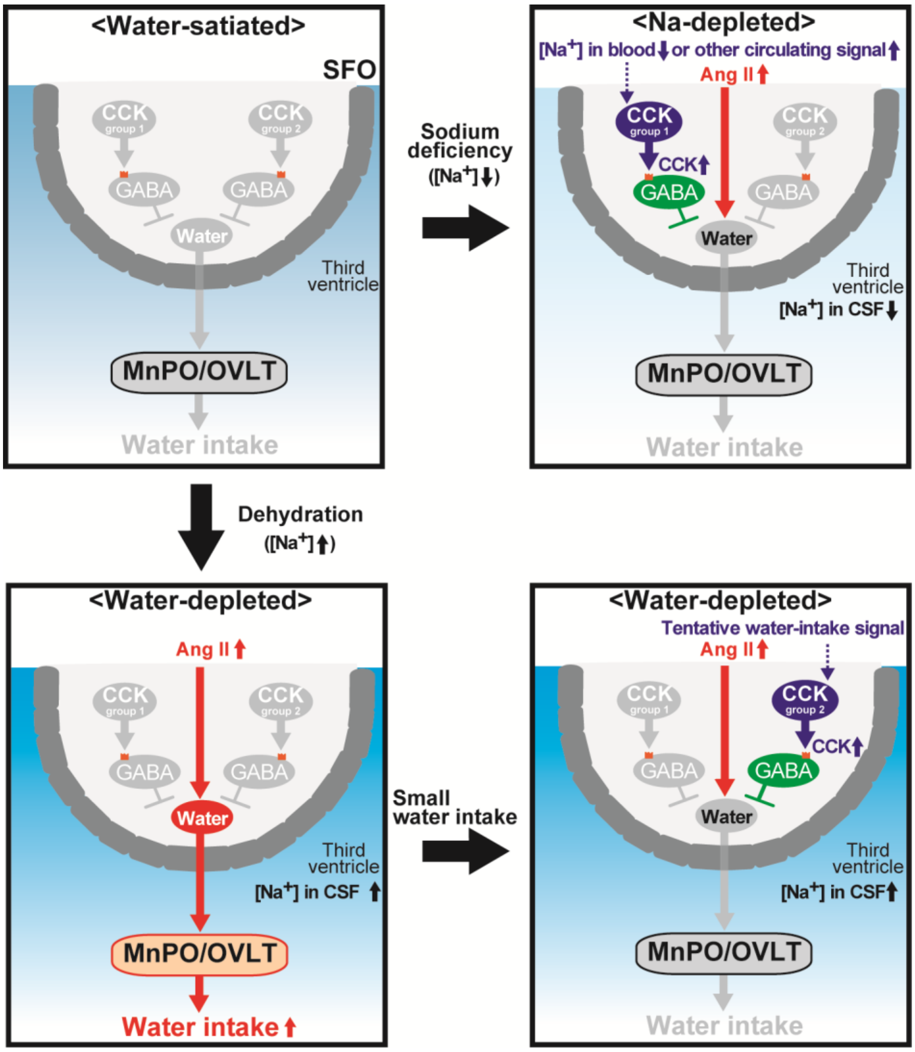

Supplementary Figure 8. Schematic drawings of neural mechanisms for the suppression of water neurons mediated by distinct CCK neurons in the SFO

The SFO deficient in the blood-brain barrier faces the third ventricle by an ependymal cell layer. Water neurons (thirst-driving neurons) in the SFO generally transmit a thirst signal to the MnPO/OVLT in order to induce water-intake behavior. The present study revealed that the SFO contains two distinct subpopulations of CCK-positive neurons (groups 1 and 2) receiving humoral or neural signals.

Under the Na-depleted condition (upper right), circulating Ang II levels increase in blood. Ang II normally stimulates water neurons in the SFO; however, water neurons are inhibited by GABAergic neurons under the Na-depleted condition. The GABAergic neurons with CCK-B receptors (indicated by red marks on GABAergic neurons) are presumably activated by CCK secreted from group 1 CCK-positive neurons in the SFO. Group 1 CCK-positive neurons may be activated by circulating

signals including [Na^+], under the Na-depleted condition. Therefore, water intake in Na-depleted mice is reduced.

Under the water-depleted condition (lower left), thirst signals such as Ang II stimulate water neurons in the SFO. When animals ingest water, other group 2 CCK-positive neurons are tentatively activated by water intake (lower right). Consequently, GABAergic interneurons in the SFO are transiently activated by CCK to suppress water neurons and stop water intake in excess. These CCK-mediated mechanisms are involved in the brain mechanisms responsible for water-intake control in order to prevent excess water intake.
