# Supplementary material for: Distinct CCK-positive SFO neurons are involved in persistent or transient suppression of water intake
Source: Nat Commun. 2020 Nov 10;11:5692. doi: 10.1038/s41467-020-19191-0 (PMC7655816; doi:10.1038/s41467-020-19191-0)
Supplement: Supplementary file 3 — Description of Additional Supplementary Files [file 41467_2020_19191_MOESM3_ESM.pdf]

Description of Additional Supplementary Files

File Name: Supplementary Movie 1
Description: Calcium imaging of CCK-positive neurons in the SFO under the Na-depleted condition and in response to salt intake.

File Name: Supplementary Movie 2
Description: Calcium imaging of CCK-positive neurons in the SFO time-locked to water intake.
