# Supplementary material for: Distinct CCK-positive SFO neurons are involved in persistent or transient suppression of water intake
Source: Nat Commun. 2020 Nov 10;11:5692. doi: 10.1038/s41467-020-19191-0 (PMC7655816; doi:10.1038/s41467-020-19191-0)
Supplement: Supplementary file 6 — Reporting Summary [file 41467_2020_19191_MOESM6_ESM.pdf]

nature research

Corresponding author(s): Masaharu Noda

Last updated by author(s): Oct 1, 2020

Reporting Summary

Nature Research wishes to improve the reproducibility of the work that we publish. This form provides structure for consistency and transparency in reporting. For further information on Nature Research policies, see Authors & Referees and the Editorial Policy Checklist .

Statistics

For all statistical analyses, confirm that the following items are present in the figure legend, table legend, main text, or Methods section.

n/a	Confirmed	
☐	☒	The exact sample size (n) for each experimental group/condition, given as a discrete number and unit of measurement
☐	☒	A statement on whether measurements were taken from distinct samples or whether the same sample was measured repeatedly
☐	☒	The statistical test(s) used AND whether they are one- or two-sided<br>*Only common tests should be described solely by name; describe more complex techniques in the Methods section.*
☒	☐	A description of all covariates tested
☐	☒	A description of any assumptions or corrections, such as tests of normality and adjustment for multiple comparisons
☐	☒	A full description of the statistical parameters including central tendency (e.g. means) or other basic estimates (e.g. regression coefficient) AND variation (e.g. standard deviation) or associated estimates of uncertainty (e.g. confidence intervals)
☐	☒	For null hypothesis testing, the test statistic (e.g. F, t, r) with confidence intervals, effect sizes, degrees of freedom and P value noted<br>*Give P values as exact values whenever suitable.*
☒	☐	For Bayesian analysis, information on the choice of priors and Markov chain Monte Carlo settings
☒	☐	For hierarchical and complex designs, identification of the appropriate level for tests and full reporting of outcomes
☒	☐	Estimates of effect sizes (e.g. Cohen's d, Pearson's r), indicating how they were calculated

Our web collection on statistics for biologists contains articles on many of the points above.

Software and code

Policy information about availability of computer code

Data collection	Conforcal images were obtained with Zeiss 2011 Imaging software, version 7.0.7.288 (Carl Zeiss).<br>Quantitation of mRNA were performed using StepOne™ and StepOnePlus™ Software (Thermo Fisher Scientific).<br>Electrophysiological data were collected using pCLAMP (Axon Instruments).<br>Fluid intake data were collected by AB-MDC (BrainScience idea).<br>Calcium imaging data were acquired with nVoke acquisition software, version 2.1.10 (Inscopix).
Data analysis	All statistical analyses were performed using Origin 2019 (Light Stone).<br>Electrophysiological data were analyzed using Clampfit (Axon Instruments).<br>Calcium imaging data were analyzed using Data Processing Software, version 1.3.0 (Inscopix).<br>ImageJ 1.48v (NIH) was used for immunofluorescence quantification of conforcal images.

For manuscripts utilizing custom algorithms or software that are central to the research but not yet described in published literature, software must be made available to editors/reviewers. We strongly encourage code deposition in a community repository (e.g. GitHub). See the Nature Research guidelines for submitting code & software for further information.

Data

Policy information about availability of data

All manuscripts must include a data availability statement. This statement should provide the following information, where applicable:

- Accession codes, unique identifiers, or web links for publicly available datasets
- A list of figures that have associated raw data
- A description of any restrictions on data availability

The data supporting our findings in this study are available from the corresponding author upon reasonable request.

Field-specific reporting

Please select the one below that is the best fit for your research. If you are not sure, read the appropriate sections before making your selection.

[x] Life sciences [ ] Behavioural & social sciences [ ] Ecological, evolutionary & environmental sciences

For a reference copy of the document with all sections, see nature.com/documents/nr-reporting-summary-flat.pdf

Life sciences study design

All studies must disclose on these points even when the disclosure is negative.

Sample size	Sample size were not predetermined, but followed previous experiments and publications (Matsuda et al., Nat. Neurosci., 2017; Zimmerman et al., Nature, 2019).
Data exclusions	Data obtained from animals in that viral infection was absent or outside the area of interest were excluded.
Replication	All experiments were performed using at least two groups of mice. All replications were successful.
Randomization	For behavioral experiments, recording chambers were randomized. In immunohistochemical studies,mice were randomly assigned to a group under control and other conditions.
Blinding	For all the behavioral experiments, blinding was not performed, because intake volumes were monitored automatically, and the experimenter was aware of the conditions being tested because the same experimenter performed both the surgical manipulation and behavioral experiments. For experiments other than behavioral studies, investigations were not blinded to the group allocation, since only one experimentor performed the data collection and analysis. However, a K-means cluster analysis are automatically performed by using Origin 2019 (Light Stone).

Reporting for specific materials, systems and methods

We require information from authors about some types of materials, experimental systems and methods used in many studies. Here, indicate whether each material, system or method listed is relevant to your study. If you are not sure if a list item applies to your research, read the appropriate section before selecting a response.

Materials & experimental systems

n/a	Involved in the study
[ ]	[x] Antibodies
[x]	[ ] Eukaryotic cell lines
[x]	[ ] Palaeontology
[ ]	[x] Animals and other organisms
[x]	[ ] Human research participants
[x]	[ ] Clinical data

Methods

n/a	Involved in the study
[x]	[ ] ChIP-seq
[x]	[ ] Flow cytometry
[x]	[ ] MRI-based neuroimaging

Antibodies

Antibodies used	Goat anti-Fos (1:500, sc-52G, Santa Cruz Biotechnology)<br>Rat anti-GFP (1:1,000, 04404-84, Nacalai Tesque)<br>Rabbit anti-RFP (1:1000, 600-401-379, Rockland)<br>Mouse anti-RFP mAb Cocktail (1:500, M208-3, MBL)<br>Rabbit ant-β-galactosidase (1:1,000, 55976, Cappel)<br>Goat anti-CCKBR (1:500, ab77077, abcam)<br>Goat anti-nNOS (1:1,000, ab1376, Abcam)<br>Rabbit anti-GFAP (1:1000, Z0334, DAKO)<br>Donkey anti-mouse IgG Alexa Fluor 555 (1:500, A-31570, Invitrogen)<br>Donkey anti-rabbit IgG Alexa Fluor 488 (1:500, A-21206, Invitrogen)<br>Donkey anti-rabbit IgG Alexa Fluor 555 (1:500, A-31572, Invitrogen)<br>Donkey anti-rat IgG Alexa Fluor 488 (1:500, A-21208, Invitrogen)<br>Donkey anti-goat IgG Alexa Fluor 488 (1:500, A-11055, Invitrogen)<br>Donkey anti-goat IgG Alexa Fluor 555 (1:500, A32816, Invitrogen)
Validation	The antibodies including anti-Fos (sc-52G), anti-GFP (04404-84), and ant-β-galactosidase (55976) were already validated in the previous study (Matsuda et al., Nature Neuroscience, 2017).<br>The antibodies of anti-RFP (600-401-379), anti-RFP Cocktail (M208-3), anti-GFAP (Z0334), and secondary antibodies were

validated by immunohistochemistry and immunocytochemistry of mouse tissues by manufacturers.
The antibodies of anti-CCKBR (ab77077) and anti-nNOS(ab1376) were validated in Immunocytochemistry (Xu L et al., Proc. Natl. Acad. Sci. U S A, 2012; Oka et al., Nature, 2015).

Animals and other organisms

Policy information about studies involving animals; ARRIVE guidelines recommended for reporting animal research

Laboratory animals	All mice with C57BL6/J background were used. Mice were housed in a temperature- and humidity-controlled room (23ºC–25 °C, 40–60%) with a 12-hr light-dark cycle (lights on at 8:00 a.m.), and were allowed free access to water and food (Rodent Diet CA-1, CLEA Japan). Experimental surgery was performed on male mice from at least 8 weeks of age. Behavioral and electrophysiological experiments were then conducted at least 4 weeks after surgery.
Wild animals	Wild animals were not used in this study.
Field-collected samples	Field-collected samples were not used in this study.
Ethics oversight	All experiments with animals were performed according to the protocols admitted by institutional animal care and use committee of National Institutes of Natural Sciences, Japan (approval numbers 17A006, 18A018, and 19A040) and Tokyo Institute of Technology (approval numbers D2019013).

Note that full information on the approval of the study protocol must also be provided in the manuscript.
